# Evaluation of a Hybrid Cardiovascular Rehabilitation Program in Acute Coronary Syndrome Low-Risk Patients Organised in Both Cardiac Rehabilitation and Sport Centres: A Model Feasibility Study

**DOI:** 10.3390/ijerph19159455

**Published:** 2022-08-02

**Authors:** Jean-Baptiste Meslet, Benoit Dugué, Ugo Brisset, Alain Pianeta, Sophie Kubas

**Affiliations:** 1 Laboratoire «Mobilité Vieillissement, Exercice (MOVE)-UR 20296», Faculté des Sciences du Sport, Université de Poitiers, 86000 Poitiers, France; jean.baptiste.meslet@univ-poitiers.fr; 2 Centre de Réadaptation Cardiaque de Bois-Gibert, 37510 Ballan-Miré, France; ugobrisset@hotmail.fr (U.B.); pianeta@orange.fr (A.P.); sophie.kubas@vyv3.fr (S.K.)

**Keywords:** acute coronary syndrome, cardiac rehabilitation, hybrid rehabilitation, physical activity medical prescription

## Abstract

The aim of the study was to investigate the efficiency, the feasibility, and the safety of a hybrid cardiovascular rehabilitation program in low-risk acute coronary syndrome (ACS) patients. Sixty low-risk patients with stable clinical status who experienced an ACS in the previous 3 months were included in a 3-week rehabilitation program. The patients were randomized either to a group performing the rehabilitation totally in a rehabilitation centre or partially (only the first 5 days) and then in sport centres equipped for supervised adapted physical activities. The sport centres were located in the vicinity of the patient’s home. Both rehabilitation programs entailed endurance and resistance training and educational therapy. Before and after rehabilitation, cardiorespiratory functions were measured. Similar and significant improvements in peak $\dot{V}$O_2_ and power output were seen in patients after both types of rehabilitation (*p* < 0.05). No particular complications were associated with both of our programs. We conclude that a hybrid rehabilitation program in low-risk ACS patients is feasible, safe, and as beneficial as a traditional program organised in a rehabilitation centre, at least in a short-term. A longitudinal follow-up should nevertheless be organised to examine the long-term impacts of this hybrid rehabilitation program.

## 1. Introduction

Survivors of a first acute coronary syndrome (ACS) deserve a specific care as the risk of experiencing a second cardiac event is significant [1]. Centre-based rehabilitation programs can be offered to such patients to lower such risk, facilitate recovery, and prevent further cardiac illnesses. The effectiveness of cardiac rehabilitation is well established and in the recent years, the prognosis of ACS has improved [2,3,4].

Nevertheless, the prescription of such rehabilitation in cardiac rehabilitation centres is rather low worldwide both in developed and less developed countries [5]. This situation stems from many different circumstances: the absence of a cardiac rehabilitation centre in the vicinity of patient’s home, patient difficulties to invest enough time and interest for their rehabilitation, and the reluctance of medical authorities to prescribe centre-based rehabilitation programs. For instance, in France, the prescription rate of cardiac rehabilitation programs is around 28.5% for patients, 6 months after a cardiac event, and fluctuates between 3.7% (female patients in the area of Martinique) to 45.3% (male patients in Centre region) depending on the regions of the country [5].

Of course, this situation is unsatisfactory. Moreover, it has been observed that prescription rate is inversely correlated with patient risk. In other terms, rather low-risk patients are more easily assigned to cardiac centres than more high-risk patients are. This situation is unfortunate since it has been shown that in a 1-year survival study after acute myocardial infarction, the greatest mortality reduction was observed in high-risk patients who followed cardiac rehabilitation in a rehabilitation centre [6]. Cardiac rehabilitation centres are clearly the best places for such patients with a 24 h surveillance with qualified personnel, adjusted medical treatment, and individualized rehabilitation.

In the context of health prevention, treatment, and rehabilitation, a key issue is to provide the best available and most cost-effective intervention. Considering precision and personalized medicine, the importance of patient profiling to identify specific needs is crucial. Such an approach has advantages as individualized intervention can be tailored to specific outcomes according to the individual capacities. Therefore, it is logical to devise different kinds of procedures for low-risk and high-risk ACS patients. The high- or higher- risk patients should have priority for care in cardiac centres.

The key issue for relevant care of low-risk ACS patients concerns the prevention of secondary cardiovascular disease through a multidisciplinary rehabilitation to reduce cardiovascular disease risk profile and improve exercise capacity. To achieve this purpose, organising the whole rehabilitation procedure in a cardiac rehabilitation centre may not be necessary. In the recent years, several alternatives have been explored. For instance, home-based or different kinds of hybrid cardiac rehabilitation have been evaluated and found to be of rather similar impact as traditional rehabilitation for different kinds of cardiac patients [4,7]. However, after a traditional or a hybrid rehabilitation, the patient tends to return to a sedentary lifestyle with too little time involved in regular exercise as compared with the recommended hours of exercise during rehabilitation [8,9]. In order to prolong the health gains made in traditional or hybrid rehabilitation, actions should be taken to motivate patients in their rehabilitation and facilitate access to supervised appropriate physical activities and educational therapy in order to induce a lifestyle change in the practice of regular physical activities for long-term healthy lifestyle changes.

Therefore, to increase access and participation to cardiac rehabilitation, to increase available options for prescribing cardiac rehabilitation, and to enhance long-term patient adherence to the program, we designed a hybrid cardiac rehabilitation model where the first stages of the rehabilitation are conducted in a traditional rehabilitation cardiac centre and the following in sport centres located in the vicinity of patient’s home under the supervision of trainers/educators who have specialised training in adapted physical activity. This study is investigated in terms of feasibility, efficiency, and safety.

## 2. Subjects and Methods

Sixty low-risk ACS patients with stable clinical status participated in two types of rehabilitation programs. The inclusion criteria were the onset of an ACS in the 3 previous months possibly treated with a percutaneous transluminal coronary angioplasty, with a low evolution risk profile (noncomplex clinical evolution during hospital stay, no recurrent ischemic heart disease, no heart failure, no severe ventricular arrhythmias, good functional capacities (>6 METS), preserved systolic left ventricular function with an ejection fraction higher than 50%, the absence of any residual myocardial ischemia at rest and during exercise, no severe ventricular arrhythmias at rest and during exercise, and an adapted blood pressure rise during exercise. Moreover, patients were over 18 years old and affiliated to the French social security system (national health insurance program). Exclusion criteria included locomotor disability, cognitive issues, or disabling disease that might interfere with the exercise protocol, all contra-indications to exercise test or training sessions, as well as abnormal symptoms during the rehabilitation process. Study protocols complied with the Helsinki declaration and was approved by the Ethics Committee of Biomedical Research (ref ID-RCB: 2018-A01587-48—CPP EST 1: 2018/52, France). All of the subjects were informed about the study procedures and gave their written consent.

### 2.1. Study Design

**Figure 1:**
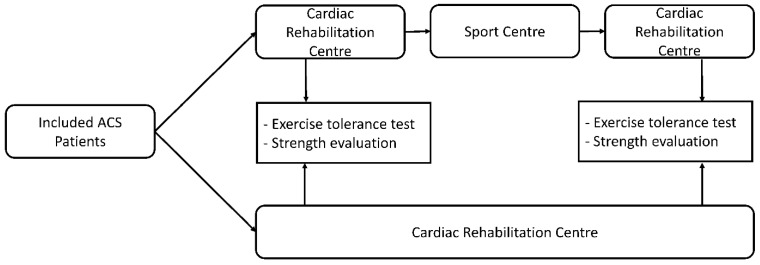
Design of the study.

The patients were referred for cardiac rehabilitation after an ACS and then randomized either to a group performing the training totally in a rehabilitation centre (3 weeks) or partially (only the first 5 days) and then during 3 weeks in sport centres equipped to supervise adapted physical activities until the end of the program (hybrid rehabilitation program). The sport centres were located in the vicinity of the patient’s home. Allocation to each group was determined by computer-generated random numbers. Demographic data were gathered before and after the traditional and the hybrid rehabilitation and included low-density (LDL) and high-density lipoprotein (HDL) cholesterol levels. Evaluations of patients were performed at the rehabilitation centre before and after both rehabilitation programs through a symptom-limited cardiopulmonary maximal exercise test and lower and upper limb strength tests (Figure 1).

### 2.2. Exercise Tolerance Test

The subjects performed a standardized symptom-limited cardiopulmonary maximal exercise test on an electrically braked stationary cycle ergometer (Ergometric 900 ERG, GE Medical System, CASE Exercise Testing System Case, Freiburg, Germany) using a ramp protocol, before and after the rehabilitation programs as previously described [10]. After a 2-min warm-up at 30 W, the workload was increased by 10 watts every minute, until the patient became exhausted. The test ended when the subject was no longer able to pedal the cycle despite encouragement. Recovery was performed without any resistance during 2 min. The subjects breathed through a facemask during the whole exercise test and exhaled gas flows were measured using a pneumotachograph and analyzed breath-by-breath using an automated system (Vmax Spectra, Sensor Medics, Yorba Linda, CA, USA). A 12-lead ECG recorded heart rate continuously (Case, GE Medical Systems Information Technologies, Milwaukee, WI, USA). Oxygen uptake ($\dot{V}$O_2_), carbon dioxide production ($\dot{V}$CO_2_), expiratory flow (VE), and standard respiratory parameters were continuously monitored and averaged every 10 s. The first ventilatory threshold (VTh1) was evaluated using the Beaver and Wasserman method [11].

### 2.3. Strength Test

After a 10-min standardized warm-up, a one-repetition maximum strength test is performed according to Jidovtseff et al. [12]. A similar procedure is used for the upper and the lower limbs.

Each patient performed a set of 10 repetitions with a weight that was approximately 30–40% of the predicted 1RM. Patients rested for 3–5 min after which a set of 4–6 repetitions was completed with a load of approximately 50–70% of the predicted 1RM. After a 3–5 min rest period, the weight was increased to an estimated 90%RM load with a single repetition. The weight was then increased until failure was reached. The heaviest successfully lifted weight was considered as the 1RM. Again, there was a 3–5 min rest period between each attempt.

### 2.4. Rehabilitation Programs

In both rehabilitation programs, the patients were followed by a multidisciplinary team with cardiologists, medical doctors, specialized trainers in adapted physical activity, and dietician specialists. In both programs, the first five days were spent in the cardiac rehabilitation centre. Patients were included in the study groups, medically checked, informed about their condition and pathology through a therapeutic education program, given recommendations for their diets, and evaluated for physical prescription through exercise tolerance and strength tests. In both programs, the first training sessions were then performed at the cardiac rehabilitation centre for the first three days. The physical activity programs combined both endurance and resistance training and the prescription defined the training intensity of the exercises through cardiac frequency targets, the corresponding power output or exercise intensity on Borg scale.

Two sessions of aerobic training were organised each day. One session consisted of exercises performed on a cycle ergometer as follows: a 5-min warm-up period followed either by 20 min of exercise at an individualized target intensity heart rate recorded at the first ventilatory threshold during the first exercise tolerance test and a 5-min period of active recovery or by 12 sequences of 30 s at 110–150% of the first ventilatory threshold workload, followed with 60 s at 40–80% of the first ventilatory threshold workload [13,14]. The second aerobic session consisted of a 1-h walk, which each patient was asked to perform. The program also included one resistance exercise session per day. The sessions began with a 5-min warm-up (slow-pace walk, segmental movements at low speed, stretching). The core of the session lasted 25 min and was devoted for at least four to seven resistance training exercises (leg press, horizontal pulling, leg extension, bench press, walking lunges, shoulder press, ankle extension) alternating lower and upper limb exercises [4,15,16,17,18]. Each exercise consisted of 1–3 series of 15–20 repetitions at a 40–60% 1RM load. Between each series, the patients rested for 1–3 min. The repetitions were organised at a load that the patients would not reach failure. The exercise intensity was monitored using a Borg scale and the patients were requested not to exceed the rating of 8/10. The total isometric training time was checked not to exceed 10% of the 30 min resistance training part. The session ended with a 5 min recovery while seated. All training sessions were conducted under the supervision of a physical therapist or an educated trainer in adapted physical activities. 

In case of an abnormal cardiac event occurring during this initial phase in patients belonging to the hybrid program, the patients were excluded from the study. At the end of this first 3-day training, an agreement contract containing individualized objectives was signed by the patient. A booklet presenting the sessions that the patients would follow in the second part of the program was also given with individualized intensity training targets. Patients or the sports centre personal trainer were asked to fill in description, duration, and intensities of each session right after each session. They were also asked to report any adverse events.

Concerning the second part of the rehabilitation program, the groups of patients continued their protocols at either the cardiac centre or in a delocalized sport centre. The patients who stayed at the cardiac centre continued the same program as described above, 5 times per week. Those who went to the sport centres were asked to follow a 3-week program with 3 sessions of 1 h per week. The session consisted of 30–40 min of aerobic exercise performed on a cycle ergometer similar to the training performed in the cardiac rehabilitation centre. The following 20–30 min were devoted to at least four to seven resistance training exercises performed in a similar fashion as the training received in the cardiac rehabilitation centre. Besides the planned session at the sport centre, the patients were also asked to go for a 1 h walk each day.

The first session at the sport centre was considered as an introductory session and was conducted by the patient personal trainer under the supervision of a member of the cardiac centre involved in the project. The following sessions were then conducted under the supervision of the patient personal trainer where the patient was continuously followed. Only one person was being supervised at a time.

At the end of the hybrid rehabilitation program, a satisfaction score was given by the patients on scale from 0 to 10 (0 being the worst and 10 the best). Patients received a booklet proposing exercises (description, duration, perceived intensity) that were recommended to be performed on daily basis.

### 2.5. Information concerning the Collaboration with Patient’s GPs during the Hybrid Rehabilitation Program and the Selection of the Sport Centres

Each patient’s personal GP was informed of his patient’s inclusion in the sport centre rehabilitation program and his collaboration was needed to ensure the quality of the rehabilitation follow-up care in case of a medical problem. Systematically, a patient appointment was scheduled with his GP during the 2nd week of the delocalized rehabilitation phase. The GP also had a direct phone access to the cardiologists and/or physical therapists of the cardiac rehabilitation centre as well as to the referred personal from the sport centre.

Before addressing participants to a sport centre, the cardiac rehabilitation centre reviewed the sport centres in its region or district. The selected sport centres were requested to have professional educators in physical adapted activities or physical trainers, established protocol in case of emergency, and an automated external defibrillator available in the room where the training sessions were organised. In order to enhance the personnel abilities to supervise low risk ACS patient exercise, a specific in-service training was organised by the cardiac centre. The training for the sports centre trainers focused on gauging and developing knowledge in cardiovascular diseases, the specific needs of low-risk ACS patients, monitoring of training, and emergency protocols in case of health problems during the training sessions.

### 2.6. Statistical Analysis

#### 2.6.1. Sample Size

To assure a non-inferiority hypothesis on the primary outcome (expected changes in $\dot{V}$O_2_ peak comprised between 3 to 5 mL/kg/min with a non-inferiority of 3 mL/kg/min), a one-sided type I error of 0.025 and 80% power, a total sample size of 42 patients (21 assigned to each group) is required. This number of recruited patients was increased to 30 in each group in order to prevent possible dropouts.

#### 2.6.2. Data Management

The data are expressed as the mean ± standard deviation. Statistical analysis was performed using Statistica software (Statsoft, Maisons Alfort, France). Kolmogorov–Smirnov and Lilliefors tests were used to check the normality of the distribution. Student’s *t* test was used to compare the baseline characteristics of the groups. We compared the data obtained before and after the two types of rehabilitation using a two-way analysis of variance for repeated measurements. The Scheffé test was used as a post hoc test when a significant interaction was observed. *p* < 0.05 was considered significant.

## 3. Results

The characteristics of the patients who completed the entire study are presented in Table 1. There were two dropouts in the hybrid program group where patients withdrew their consent and refused to further participate in the study.

The main changes observed in both programs are reported in Table 2. Significant improvements (*p* < 0.05) in $\dot{V}$O_2_ and power output peaks as well as in peak heart rate and power output at the first ventilatory threshold were observed. No significant changes in upper and lower limb strength were noted. The patient serum HDL-cholesterol concentration stayed stable in both groups when comparing the data obtained before vs. after the rehabilitation (0.41 ± 0.10 vs. 0.40 ± 0.10 and 0.38 ± 0.10 g/L vs. 0.38 ± 0.10 g/L, *p* < 0.05, in the traditional and in the hybrid rehabilitation groups, respectively). Concerning the changes in LDL-cholesterol, we observed a significant decrease in both groups when comparing the data obtained before vs. after both rehabilitations (0.81 ± 0.39 vs. 0.57 ± 0.36 and 0.66 ± 0.21 g/L vs. 0.56 ± 0.17 g/L, *p* < 0.05, in the traditional and in the hybrid rehabilitation groups, respectively). However, the changes were considered similar in both groups as no significant interaction was found. No adverse events were noted during the hybrid rehabilitation program. All the participants from this program performed the 1 + 9 scheduled sessions that were planned. Those patients also gave a satisfaction score of 9.9/10 for their rehabilitation. From the 28 participants belonging to the hybrid program, 18 of them continued regular visits to the sport centre after the rehabilitation program officially ended.

## 4. Discussion

**Figure 2:**
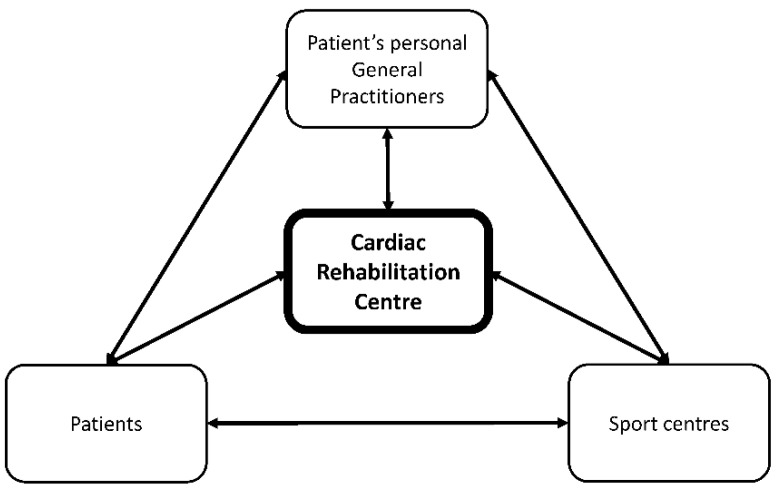
Organization of the hybrid cardiac rehabilitation model where the cardiac rehabilitation centre acts as the control tower of the whole rehabilitation.

In this preliminary trial, we developed a concept of a new hybrid cardiovascular rehabilitation program where the cardiac rehabilitation centre is kept as the control tower of the whole rehabilitation processes but where a part of the rehabilitation is performed outside of its walls (Figure 2). This model here is designed for low-risk stable patients after an acute coronary syndrome. The idea is that low-risk patients in general do not need complete medical assistance/surveillance during a rehabilitation program and that a part of it can be organized elsewhere than in a rehabilitation centre (in the present study, in sport centres located in the vicinity of patient’s home). This new program is organised with a stay for few days (in the present study, 5 days) in the cardiac rehabilitation centre where patients are evaluated, educated on their pathology, given health-related information and how to function in daily life, and given an individualized training protocol with specific aims for the rehabilitation in terms of amount, intensity, type, duration, and frequency of physical activities. The first sessions of the rehabilitation program are given at the rehabilitation centre. Then, the following part of the program (3 weeks) is proposed in specific sport centres where the personnel are educated to supervise heart disease patients’ physical activities (e.g., trainer specialised in adapted physical activity). The sport centres are first selected by the rehabilitation centre through a series of criteria, and the rehabilitation centre provides extra tuition for patient care. To effectively implement this hybrid model of cardiac rehabilitation, in-depth collaborations and communications need to be organised between the cardiac rehabilitation centre in charge of the patient’s rehabilitation, the sport centres with their adapted physical activity trained specialists, associated dieticians, and the patient’s general practitioner. 

This model offers several potential advantages and could, at the same time, lower the medical cost dedicated to traditional cardiac rehabilitation. The savings could then be re-invested for the treatment of more severe ACS patients and allow more ACS patients access to cardiac rehabilitation programs after a cardiac event. This hybrid model could also facilitate the everyday life of low-risk ACS patients who are unable to stay, visit, or reach rehabilitation centres for various reasons; increase the amount of physical activity in patients; improve the patient satisfaction and adhesion to the proposed rehabilitation program; help patients incorporate sports centre visits into daily life; help patients incorporate long-term healthy lifestyle changes concerning regular physical activity; and improve the quality of life in patients.

In this time of precision and personalized medicine, such a tailored approach needs to be first investigated in terms of feasibility, efficiency, and safety. The main results of this investigation are that it is possible to safely organize such a rehabilitation program since we did not observe any special complications during the length of the study, and it seems to also be as effective as a traditional rehabilitation, at least in a short-term. We found that the changes in $\dot{V}$O_2_ and power output peaks were similar between this hybrid model and regular rehabilitation and consistent with the outcomes published in the scientific literature [10,19]. Concerning the significant decrease in LDL-cholesterol blood concentration, again the changes were quite similar in both programs. An added benefit to the hybrid model was the extremely high rate of patient satisfaction (9.9/10) indicating a good combination of each patient’s physical activity rehabilitation needs and the patient’s understanding and fulfilment of instructions needed to complete the hybrid model training regime.

Patient adhesion to the hybrid program was also high as no patients dropped out of the study in the second part of the program and all patients participated in every planned rehabilitation session. Another indicator of patient adhesion is that 18 of the 28 participants in the hybrid rehabilitation group continued to visit the sport facilities on regular basis after the study was completed.

There have been many initiatives around the world to study alternatives to traditional cardiac rehabilitation in specialized rehabilitation centres. Such hybrid cardiac rehabilitations offer a mix of direct supervised centre-based and home-based cardiac rehabilitation with the use of special communication devices (e.g., remote systems, visio-systems, and tele-rehabilitation) between patients and health/medical supports. A recent meta-analysis concluded that hybrid cardiac rehabilitation globally showed similar efficacy to the traditional model, at least in the short term. However, the long-term outcomes are for the time being less clear and still need to be investigated [7,20,21]. 

Some recent cost-efficient care delivery strategies using hybrid rehabilitation claimed to induce long-term health benefits. However, even though such long-term health benefits were shown, the authors nevertheless expressed that a significant stronger decline in $\dot{V}$O_2_ peak was observed in patients after hybrid rehabilitation compared to those following the traditional one [22]. However, whether one kind of rehabilitation is better than the other one in the long-term may not be a key issue as both of the approaches share major drawbacks with low adhesion, high sedentary behaviour, and low physical activity levels [8,9]. 

Our proposed individual tailored intervention focused on supervised adapted physical activity performed in specific sport centres in the vicinity of the patients’ living places. Such intervention is characterised by a reduced fee for medical supervision, is more practical for the patient who can more easily combine the rehabilitation with his/her own agenda. This aspect may help for program adhesion and long-term behavioural change. The proposed hybrid intervention is also in line with the recent possibilities of medically prescribed physical activities for chronic disease patients. This approach is rather new, and the patient’s service offers need to be developed both in France and in other parts of the world. Our hybrid intervention model could serve such a purpose and could even be generalised to any kind of low-risk patients (not only cardiac patients but also those with other pathologies such as respiratory diseases, low-back pain, etc.). In rehabilitation centres, low-risk patients could be included on Mondays, instructed, and trained in the physical exercise programs and to the different components of the traditional rehabilitation. Then, if no difficulties or medical problems occur, they could be discharged on Fridays to pursue their rehabilitation using this hybrid model. A follow-up check-up visit could be scheduled at the traditional rehabilitation centre to measure progress after the program and/or at distance of the program.

## 5. Conclusions

In conclusion, a new hybrid cardiac rehabilitation program in low-risk ACS patients —where the first part of the rehabilitation is organised in a cardiac rehabilitation centre and then in habilitated sport centres located near the patient’s home—is feasible, safe, and as beneficial as a traditional program totally organised in a rehabilitation centre, at least in a short-term. However, the present investigation is still at its preliminary phase, and the obtained results need to be confirmed with a larger number of patients and with a precise follow-up to investigate the long-term outcomes of such approach.

## Figures and Tables

**Table 1:** Patient characteristics at baseline.

	Centre-Based Rehabilitation n = 30	Hybrid Rehabilitation n = 28
Age (years)	57 ± 10	56 ± 9
Height (cm)	171 ± 6	171 ± 7
Weight (kg)	84 ± 17	75 ± 12 ^#^
BMI (kg.m^−2^)	28 ± 5	26 ± 4 ^#^
Waist size (cm)	101 ± 14	96 ± 10
LVEF (%)	57 ± 5	57 ± 5
$\dot{V}$O_2_ peak (mL/min/kg)	21 ± 6	23 ± 4
HDL (g/L)	0.41 ± 0.10	0.38 ± 0.10
LDL (g/L)	0.81 ± 0.39	0.66 ± 0.21
Framingham score %	9 ± 8	10 ± 7
Smokers (n)		
No	11	7
Active	8	13
Former	11	8
Associated pathologies		
Diabetes	4	5
Dyslipidemia	14	14
Cardiac family history	19	18
Treatments		
Beta blockers	29	28
Antiplatelets	29	28
Vasodilators ^a^	30	28
Statins	27	28

Abbreviations: BMI = body mass index; LVEF = left ventricular ejection fraction; $\dot{V}$O_2_ peak = oxygen consumption at peak exercise tolerance test; ^a^ Angiotensin-converting enzyme inhibitors and angiotensin receptors-II antagonists. ^#^ Significantly different from centre-based rehabilitation group (*p* < 0.05).

**Table 2:** Cardiopulmonary variables during exercise tolerance test and the maximum amount of force generated in one maximal contraction test.

	Before Rehabilitation		After Rehabilitation	
	Centre-Based Rehabilitation n = 30	Hybrid Rehabilitation n = 28	Centre-Based Rehabilitation n = 30	Hybrid Rehabilitation n = 28
$\dot{V}$O_2_ peak (mL/min/kg)	21 ± 6	23 ± 4	24 ± 6 *	26 ± 4 *
Peak power output (W)	134 ± 27	135 ± 32	158 ± 33 *	161 ± 38 *
Power output at VT (W)	73 ± 20	80 ± 20	88 ± 26 *	90 ± 28 *
Peak heart rate (bpm)	125 ± 21	125 ± 20	131 ± 19	132 ± 18
Lower limb 1-RM (kg)	131 ± 43	126 ± 35	144 ± 55	139 ± 43
Upper limb 1-RM (kg)	25 ± 9	29 ± 8	30 ± 10	30 ± 8

Abbreviations: * significantly different from the data obtained before rehabilitation, *p* < 0.05; $\dot{V}$O_2_ peak = oxygen consumption at peak exercise tolerance test; VT = first ventilatory threshold; bpm = beats per minute; 1-RM = one-repetition maximum.

## Data Availability

The collected data are stored at the Centre de réadaptation cardiaque de Bois-Gibert, Ballan-Miré, France.
